# An integrated workflow for real-time chemoenzymatic CMP-sialic acid preparation and a bioorthogonal microplate sialyltransferase assay (MPSA)

**DOI:** 10.1093/biomethods/bpag058

**Published:** 2026-10-14

**Authors:** Lucas Lancel, Sushmaa Dangudubiyyam, Clémence Simon, Hugo Segura, Christophe Biot, Yann Guérardel, Anne Harduin-Lepers, Cédric Lion

**Affiliations:** Univ. Lille, CNRS, UMR 8576 - UGSF - Unité de Glycobiologie Structurale et Fonctionnelle, Lille, France; Univ. Lille, CNRS, UMR 8576 - UGSF - Unité de Glycobiologie Structurale et Fonctionnelle, Lille, France; Univ. Lille, CNRS, UMR 8576 - UGSF - Unité de Glycobiologie Structurale et Fonctionnelle, Lille, France; Univ. Lille, CNRS, UMR 8576 - UGSF - Unité de Glycobiologie Structurale et Fonctionnelle, Lille, France; Univ. Lille, CNRS, UMR 8576 - UGSF - Unité de Glycobiologie Structurale et Fonctionnelle, Lille, France; Univ. Lille, CNRS, UMR 8576 - UGSF - Unité de Glycobiologie Structurale et Fonctionnelle, Lille, France; Univ. Lille, CNRS, UMR 8576 - UGSF - Unité de Glycobiologie Structurale et Fonctionnelle, Lille, France; Univ. Lille, CNRS, UMR 8576 - UGSF - Unité de Glycobiologie Structurale et Fonctionnelle, Lille, France

**Keywords:** Sialyltransferase assay, CMP-sialic acid, 31P NMR, bioorthogonal microplate assay, Chemo-Enzymatic Synthesis, Inhibitor Screening

## Abstract

Sialylation at the terminal ends of cell-surface glycoproteins and glycolipids plays a pivotal role in dictating cellular interactions, human health, and pathological processes. This crucial modification is mediated by a family of 20 distinct human sialyltransferases (STs) whose exquisite substrate specificities and stereochemistry make them high-priority therapeutic targets. Consequently, robust techniques to detect and quantify ST activity are critical for translational research and inhibitor screening. To address this need, we present a detailed protocol for the chemo-enzymatic synthesis of a C5 alkyne-functionalized CMP-Sia donor substrate tailored for bioorthogonal click chemistry. This synthesis is coupled with the development of a simple, highly sensitive, and reproducible microplate-based assay designed to perform in-depth enzymatic characterization of individual ST activities. The protocol eliminates common throughput limitations by streamlining substrate preparation and optimizing microplate detection metrics. Our results demonstrate that this assay provides exceptional sensitivity and consistency across diverse ST isoforms. Ultimately, this unified method establishes one of the most robust, easily replicable, and high-throughput platforms currently available for ST profiling and small-molecule inhibitor screening.

## Introduction

Sialic acids (Sia) are nine-carbon monosaccharides derived from neuraminic acid (Neu, 5-amino, 3,5-dideoxy-D-glycero-D-galacto-nonulosonicacid) containing a carboxyl group at the C2 carbon, a glycerol-like three-carbon side chain at the C6 carbon, and an amino-acyl group or hydroxyl group at the C5 carbon [1, 2]. In nature, Sia may be substituted with various chemical groups like glycolyl (Gc), acetyl (Ac), methyl (Me), lactyl, phosphate, and sulfate groups, generating a family of over 50 structurally different members, which include derivatives of *N*-acetylneuraminic acid (Neu5Ac), *N*-glycolylneuraminic acid (Neu5Gc), and ketodeoxynonulosonic acid (Kdn, 3-deoxy-non-2-ulosonic acid), which are the main Sia encountered [3].

Sialylation of proteins and lipids with Neu5Ac is an essential biological modification that profoundly influences cellular recognition, signaling, and various pathological states including cancer and infectious diseases in human tissues. For example, tumor cells often overexpress sialylated glycans, which promote adhesion and migration through interactions with selectins on endothelial or immune cells. In parallel, binding inhibitory Siglecs can suppress immune cell activation and reduce phagocytosis [4, 5]. Together, these mechanisms support tumor progression and metastasis [6, 7]. In infectious diseases, pathogens like influenza viruses and bacteria such as serogroup B *Neisseria meningitidis* or strains of *Escherichia coli* K1 associated with neonatal meningitis or uropathologies exploit sialylation to evade immune detection or adhere to host cells [8].

Sialyltransferases (STs) [9] catalyze the transfer of Sia from activated cytidine monophosphate derivatives (CMP-Sia) to the terminal positions of glycans [10]. The human genome encodes twenty distinct ST genes [11]. Based on their specific monosaccharide acceptors and the type of glycosidic linkage formed (α2,3-, α2,6-, or α2,8-), these enzymes are classified into four distinct families: ST3GAL (ST3GAL1-ST3GAL6), ST6GAL (ST6GAL1 and ST6GAL2), ST6GALNAC (ST6GALNAC1–ST6GALNAC6), and ST8SIA (ST8SIA1–ST8SIA6) [3, 11]. Investigating these sialylation pathways remains highly challenging. This difficulty stems not only from the vast structural diversity of Sia and their complex glycan acceptors, but also from the inherent instability and limited availability of modified CMP-Sia donor substrates.

Traditional methods for the preparation of both natural and modified CMP-Sia require tedious and cumbersome purification steps (most commonly combinations of anion exchange chromatography and size exclusion chromatography), leading to their rapid degradation and short shelf-life [12], which hinders their reproducible use in biochemical studies. It is indeed difficult to assess the purity grade of the CMP-Sia (commercial or synthesized) after a period of storage, therefore introducing uncertainty in its actual concentration. More importantly, the CMP by-product originating from CMP-Sia hydrolysis is a known inhibitor of STs [13], thereby introducing a potential bias on enzymatic activities measured during the assay.

Methods for evaluating ST activity present distinct trade-offs. Radioactivity-based assays offer high sensitivity but are costly, generate hazardous waste, and require specialized expertise [14, 15]. Conversely, non-radioactive assays often couple glycosyltransferases to auxiliary enzymes—such as phosphatases—to measure kinetics indirectly, which can compromise measurement accuracy [16]. While glycan-based microarrays excel at determining acceptor specificity by immobilizing glycans for probe-based detection, they are ill-suited for real-time kinetic analysis.

To overcome these limitations, we previously reported an efficient protocol for the direct synthesis of chemically modified CMP-sialic acid derivatives monitored by real-time ^31^P nuclear magnetic resonance (^31^P NMR) spectroscopy, a common technique available on the majority of NMR spectrometers, which enables consistent and highly reproducible synthesis of modified CMP-Sia substrates [17]. The synthesized CMP-sialic acids are obtained directly in pure form in 1–2 h without the need for any chromatographic purification, thereby allowing immediate use in biological assays [17]. This method yields both natural and modified CMP-sialic acids in pure form without requiring chromatographic purification, enabling immediate deployment in biological assays. Building on this foundation, we developed a robust, highly adaptable Microplate-based Assay of Sialyltransferase Activity, termed “MPSA” [18]. This versatile approach pairs alkyne- or azide-functionalized CMP-sialic acid donors closely mimicking the natural CMP-Sia donor with bioorthogonal chemistry to introduce diverse molecular probes, including fluorophores and biotin tags, after the sialylation reaction.

This platform, applicable to 13 different STs [19, 20], significantly streamlines high-throughput profiling of ST kinetics, substrate specificities, and inhibitor screening. The robustness of the MPSA workflow has been validated across diverse functional studies of animal sialylation [19–21]. Given the increasing demand for accessible synthetic approaches enabling direct bioanalytical applications, we present a fully integrated protocol combining efficient preparation of modified CMP-Sia donors and bioorthogonal microplate-based assays, designed to facilitate studies of sialylation processes and their biological implications. The protocol thus represents a powerful and user-friendly methodological advancement for glycosciences, designed to accelerate the study of complex sialylation processes.

## Protocol

### Synthesis of cytidine 5′-monophosphate-*N*-pentynoyl neuraminic acid (CMP-Neu5Al)

*Note: All equipment and materials specified in this protocol represent the exact items used in our study. Functionally equivalent instruments, reagents, or products from other suppliers may be substituted without impacting the results. A general scheme of CMP-Neu5Al synthesis is shown in* *Fig. 1*.

**Figure 1:**
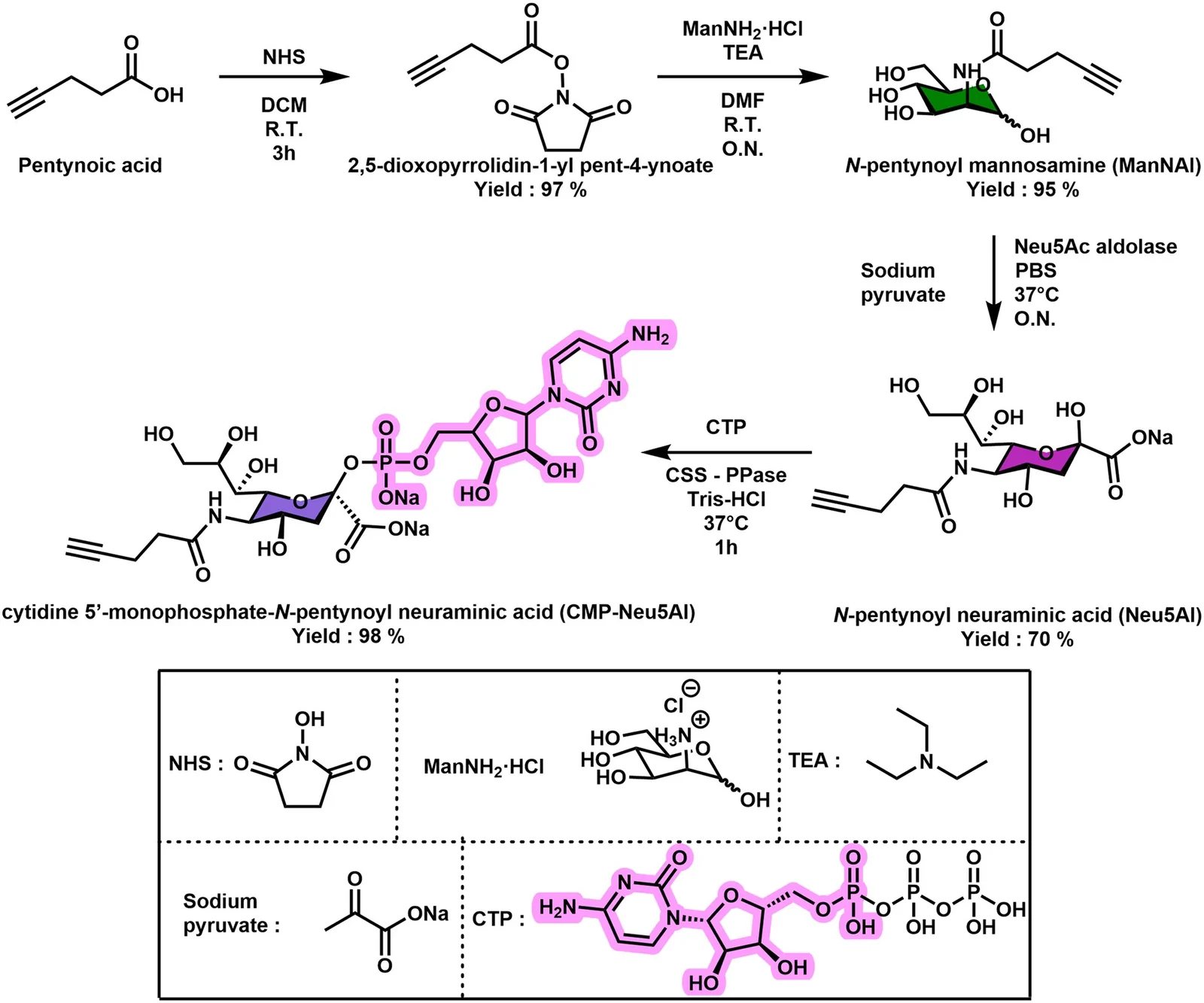
General scheme of CMP-Neu5Al synthesis. The carboxylic acid is first activated as an NHS ester. This activated ester is then used to synthesize *N*-pentynoylmannosamine (ManNAl). ManAl is subsequently reacted with pyruvate in an enzymatic reaction to produce the alkyne-modified 5-*N*-pentynoyl neuraminic acid (Neu5Al). Finally, this compound undergoes a second enzymatic reaction with CTP to yield the final CMP-sialic acid derivative. R.T. = room temperature; O.N. = overnight.

#### Preparation of synthetically modified *N*-pentynoyl mannosamine (ManNAl)

Reagents

- *N*-Hydroxysuccinimide (Merck, catalog no. 130672-100G),
- 1-ethyl(3-dimethylaminopropyl)carbodiimide (EDCI-HCl) (Merck, catalog no.^.^ E6383-25G),
- 4-pentynoic acid (Merck, catalog no. 232211-5G),
- Dichloromethane (CARLO ERBA, catalog no. 528372),
- D-mannosamine-HCl (TCI, catalog no. M3237),
- Dimethylformamide (DMF) (CARLO ERBA, catalog no. P0343522),
- Triethylamine (Merck catalog no. 471283-500ML), and
- CHROMABOND Flash SiOH 40-63 µM (machery-nagel ref no. 732803).

Equipment setup

- Round-bottom flask,
- Heathrow magnetic stirring bar,
- Buchner funnel,
- Magnetic stirrers,
- Balance,
- Separating funnel,
- Rotary evaporator,
- Fume-hood, and
- Puriflash-CMS.

Methods

*Note: Steps 1.a. and 1.b. of the synthesis should be done under fume-hood.*

1.a. Preparation of succinimidyl 4-pentynoate

- In a round-bottom flask with a magnetic stirring bar, dissolve 540 mg of 4-pentynoic acid (5.5 mmol, 1.0 eq.) in 25 mL of dichloromethane.
- Slowly add 760 mg of *N*-hydroxysuccinimide (6.6 mmol, 1.2 eq.) and 2110 mg of 1-ethyl-3-(3-dimethyl aminopropyl) carbodiimide hydrochloride (11 mmol, 2.0 eq.) under stirring.
- Allow the mixture to react for 3 h 30 min at room temperature. Monitor the reaction by TLC (cyclohexane/ethyl acetate [70:30]). R_f_ of succinimidyl 4-pentynoate: 0.74; R_f_ of pentynoic acid: 0.46).
*Note: See expected TLC profile in Fig. S1.A*.
- After completion, wash the reaction mixture four times with an aqueous 2.8% KHSO_4_ solution.
- Separate the organic layer, dry it over anhydrous Na_2_SO_4_, filter through a Buchner funnel, and evaporate the solvent under reduced pressure. The product, succinimidyl 4-pentynoate, is obtained as a pale solid (1040 mg, 5.3 mmol, 97% yield) and is used without further purification. Store it at −20°C.

Note: The NMR signals expected to confirm the chemical identity of succinimidyl 4-pentynoate are provided in the supporting information.

1.b. Synthesis of N-(4-pentynoyl) mannosamine (ManNAl)

- In a dry round-bottom flask, weigh 759 mg of D-mannosamine·HCl (3.5 mmol, 1.0 eq.) and 688 mg of succinimidyl 4-pentynoate (3.5 mmol, 1.0 eq.).
- Flush the flask with argon and dissolve the reagents in 25 ml of DMF.
- Slowly add 1.4 ml of triethylamine (10.0 mmol, 2.9 eq.) while stirring.
- Allow the mixture to stir O.N. at room temperature under argon. Monitor the reaction with TLC (propan-1-ol/20% ammonia/H_2_O (6: 1: 2.5). Rf of D-mannosamine·HCl: 0.56; Rf of N-pentynoyl mannosamine: 0.87).
  Note: See expected TLC profile in Supplementary Fig. S1.B.
- After completion, remove DMF under reduced pressure.
- Purify the crude residue by flash chromatography on silica gel (40 g, 40–63 µm), eluting with CH_2_Cl_2_/EtOAc/MeOH (45:45:10).
- Remove the solvents under reduced pressure to yield *N*-(4-pentynoyl) mannosamine (ManNAl) as a white crystalline solid (830 mg, 3.34 mmol, 95% yield). Store it at −20°C.
  Note: The NMR signals expected to confirm the chemical identity of ManNAl are provided in the supporting information.

#### Preparation of synthetically modified *N*-pentynoyl neuraminic acid (Neu5Al)

Reagents

- Phosphate-buffered saline (PBS) 1 × (EUROMEDEX catalog no. ET330),
- Sodium pyruvate (Merck, catalog no. P2256-5G),
- Sialic Acid Aldolase (Biosynth, catalog no. MS11080, *E. coli K12 BL21*, EC 4.1.3.3),
- ManNAl (synthesized in step I. 1.a), and
- NaOH 1 M (ACROS ORGANIC, no. 447280050).

Equipment setup

- Drying oven,
- Pyrex® glass culture tubes with a cap (Merck, catalog no. Z653578-40EA),
- pH-indicator paper (Sigma Aldrich, catalog no. 1109620003),
- Microtips,
- Micropipettes,
- Balance,
- Preparative HPLC with ELSD and UV detector,
- Amino column GIST 5 µm ø, 10 × 250 mm (Shimadzu, catalog no. 227-30305-04),
- Rotary evaporator, and
- Freeze dryer.

Enzyme stock

Sialic acid aldolase is weighted to have multiple aliquots of 10 mg of enzyme, dissolved in 1 ml PBS (10 U/mg; 10 mg/ml; 0.1 U/µl) when used.

Methods

Synthesis of Neu5Al

- In a glass reaction tube, dissolve 83.0 mg of ManNAl (334.0 µmol, 1.0 eq.) and 73.5 mg of sodium pyruvate (668.0 µmol, 2.0 eq.) in 1.5 ml of PBS.
  Precaution: as ManNAl is very viscous, weigh it rapidly after removing it from the −20°C freezer.
- Adjust pH to 7.5 with NaOH using pH-indicator paper,
- Add 200 µl of sialic acid aldolase (20 U),
- Adjust final volume to 2 ml by adding 300 µl of PBS.
- Incubate the tube at 37°C and stir (140 rpm) O.N.
- Monitor the reaction completion by TLC (propan-1-ol/20% ammonia/H_2_O (6: 1: 2.5)). R_f_ of *N*-pentynoyl neuraminic acid: 0.65; R_f_ of *N*-pentynoyl mannosamine: 0.87). Note: See expected TLC profile in Supplementary Fig. S1.C.
- When the reaction is over, purify the crude product on a preparative HPLC equipped with a preparative amino-column (Shim-pack GIST NH2, 5 µm ø, 10 × 250 mm). The mobile phase A is acetonitrile and the mobile phase B is water. The elution method uses a gradient starting at 10% B for 20 min, 10%–15% B in 1 min, 15% B for 20 min, 15%–90% B in 10 min, 90% B for 5 min, and 10% B for 4 min at a flow rate of 10 ml/min. Detect the product using both UV detector at 215 nm and ELSD detector. See the expected chromatogram profile in Fig. S2.
- Remove organic solvent under reduced pressure to left only water, then freeze-dry water to obtain Neu5Al as a white powder (86.3 mg, 234.0 µmol, 70% yield). Store it at −20°C.
  Note: The NMR signals expected to confirm chemical identity of Neu5Al are provided in the supporting information.

#### Preparation of synthetically modified cytidine 5′-monophosphate-5-*N*-pentynoyl neuraminic acid (CMP-Neu5Al)

Reagents

- Tris–HCl (Merck, catalog no. 10812846001),
- Cytidine 5'-triphosphate disodium salt hydrate (CTP) (Biosynth, catalog no. NC03860),
- MgCl_2_ (Merck, catalog no. M8266-100G),
- Deuterium Oxide (Eurisotop, catalog no. D216),
- Cytidine 5′-monophosphate sialic acid synthetase (CSS, Merck, catalog no. C199910UN, *Neisseria meningitidis*, EC 2.7.7.43),
- Inorganic Pyrophosphatase (0.1 U/µl) (ThermoFischer, catalog no. EF0221, *Saccharomyces cerevisiae*, EC 3.6.1.1, 1 U),
  Precaution: Keep enzymes (CSS and pyrophosphatase) on ice all along the experiment to prevent degradation.
- Neu5Al (synthesized in step I. 2.),
- NaOH 1 M (ACROS ORGANIC, no. 447280050),
- Ultra-pure water.

Equipment setup

- Pasteur pipette 350 mm (Hilgenberg, catalog no. 3150166),
- NMR Tube 5 mm ø (Brüker, catalog no. 34205723),
- Brüker 400 MHz SB Advance II spectrometer (Brüker Biospin SA, Wissembourg, France) equipped with a BBO probe (BB/^1^H/^2^H),
- pH-indicator paper (Sigma Aldrich, catalog no. 1109620003),
- Microtips,
- Micropipettes, and
- Balance.

Preparation of reaction buffer (100 ml of Tris–HCl 100 mM, MgCl_2_ 20 mM, and 10% D_2_O)

Dissolve 1.21 g Tris–HCl and 0.41 g MgCl_2_ in ultra-pure water. Adjust pH to 8.8 with NaOH. Add 10 ml of D_2_O. Complete with ultra-pure water until 100 ml.

Enzyme stock solution

CSS and pyrophosphatase are dissolved in the reaction buffer at concentration of 0.1 U/µl, aliquoted into 20 µl tubes, and stored at −20°C.

Methods

3.a. Preparation of the reactant

- In a first 1.5 ml Eppendorf tube, dissolve 8 mg of *N*-pentynoyl neuraminic acid (Neu5Al, 369.10 g/mol) in 100 µl of reaction buffer.
  Precaution: as Neu5Al is very viscous, weigh it rapidly after removing it from the −20°C freezer.
- In a second 1.5 ml Eppendorf tube, dissolve 11.42 mg of CTP (527.12 g/mol) in 100 µl of reaction buffer.
- Transfer the 100 µl of CTP solution into the Eppendorf containing the Neu5Al solution.
  Precaution: Avoid introducing bubbles at any pipetting step, as this may lead to sample loss.
- Adjust the pH to 8.8 with NaOH 1M.
  Precaution: Add base slowly (4–5 µl at a time) to avoid overshooting.
- Add the appropriate volume of reaction buffer to yield a final concentration of 34.5 mM for Neu5Al and CTP, respectively (final volume should be between 600 and 650 µl).
- Transfer the solution into an NMR tube using an extra-long Pasteur pipette and keep the tube on ice.
  Precaution: Avoid leaving solvent or buffer droplets on the inner wall of the NMR tube when filling. Ensure all liquid reaches the bottom of the tube to prevent sample loss and concentration errors.

3.b. Reaction monitoring by 31P NMR spectroscopy

^31^P NMR experiments should be acquired using the zg pulse sequence with the following parameters:

- Relaxation time (d1) of 3 s,
- Number of scans (NS) equal to 64,
- Spectrum resolution (TD) of 8192,
- Spectral window (SW) of 100,
- Center of the spectral window (O1P) of 0, and
- Temperature of 310°K (optimum temperature for CSS and PPase activity).

Wait for the spectrometer to reach temperature of 310°K.

3.c. Enzyme addition and reaction start

- Insert the experimental NMR tube into the NMR spectrometer,
- Acquire the initial spectrum corresponding to reaction time *t* = 0 min,
- Remove the tube from the instrument and transfer the solution from the NMR tube back into a 1.5 ml Eppendorf tube, and
- Add 3 µl of CSS enzyme and 5 µl of inorganic pyrophosphatase to start the reaction. Mix by gently pipetting up and down.
  Note: You can sometime notice a white precipitate inside the NMR tube. This can be a small amount of enzyme precipitating and does not influence the reaction in any sort.
- Return the solution to the NMR tube and reinsert it into the NMR spectrometer. Do not shake the NMR tube to avoid later loss of product,
- Upon addition of the CSS, continuously acquire 64-scan spectra, providing quantitative data of the conversion to CMP-Sia at each time-point of the reaction. The duration of each NMR spectrum is 3 min 43 s, and
- When the spectrum shows complete disappearance of CTP (end of the reaction) or the appearance of a new singlet peak corresponding to CMP (indicating CMP-Neu5Al degradation), remove the tube from the instrument.

Note: this real-time ^31^P NMR monitoring method can be applied to follow synthesis of other natural and chemically modified CMP-sialic acid derivatives like CMP-Neu5Az, CMP-Neu5Gc and CMP-Kdn (See Supplementary Fig. S3).

3.d. Product storage

- Aliquot the synthesized CMP-Neu5Al into 65 µl aliquots and store them at −80°C.

#### MPSA

A sialylation reaction is comprised of: (1) an ST enzyme that catalyses this reaction, (2) a nucleotide activated donor to donate the sugar, and (3) an acceptor (glycoprotein or glycolipid) onto which the sugar is transferred.

The assay is performed on a 96-well microplate, wherein the microplate surface is saturated with the acceptor glycoprotein, thus immobilizing it. Several glycoproteins, either *N*- or/and *O*-glycoproteins, sialylated or de-sialylated like fetuin, ALCAM, or porcine gastric mucin (PGM) have been used with MPSA [18, 19]. A modified sialic acid moiety (Neu5Al or Neu5Az) is transferred onto this glycoprotein. The modification on the sugar enables its detection using click chemistry. The ‘clicked’ conjugate is then linked to an antibody conjugated with a peroxidase. Tetramethyl benzidine (TMB), a chromogenic substrate, is then used to obtain the readout at 620 nm (with a standard spectrophotometer). The output is therefore a highly sensitive colorimetric reading that depends on the amount and rate of sialic acid derivative transferred onto the glycoprotein. This forms the basis of the assay, which is described below for ST6GAL1, an enzyme that sialylates galactose residues found on *N*-glycans.

Reagents

- NaHCO_3_ (Riedel de Haen, catalog no. 13433),
- Asialofetuin (Fetuin: Sigma catalog no. F2379),
- Bovine serum albumin (BSA) oxidised—[22] (Roche catalog no. 10735086001),
- Recombinant human ST6GAL1 – (R&D Systems catalog no. 7620-GT-010),
- CMP-Neu5Al (synthesized in step I.3.),
- 2.5× sodium cacodylate buffer pH 6.4,
- CuSO_4_ (Sigma catalog no. C1297),
- BTTAA (2-(4-((bis((1-(tert-butyl)-1H-1,2,3-triazol-4-yl)methyl)amino)methyl)-1H-1,2,3-triazol-1-yl)acetic acid) – (Jena BioScience catalog no. CLK-067-100),
- Sodium ascorbate (Sigma catalog no. A7631),
- Azido-biotin—(Sigma Aldrich catalog no. 762024),
- Antibody–anti-biotin conjugated with HRP—Ozyme Jackson Immunoresearch (200-032-211),
- 3,3′,5,5′—TMB—(Sigma Aldrich catalog no. T8665),
- 1× PBS (EUROMEDEX catalog no. ET330),
- 1× PBST (0.05% tween-20 EUROMEDEX catalog no. 2001-B).

Note: Depending on the enzyme used, the acceptor, donor, and time of reaction vary. The rest remains the same (see Table 1). All the recombinant sialyltransferases mentioned in Table 1 originate from the same Bio-Techne supplier (R&D Systems, Europe). The references of these products are: ST3Gal I (6905-GT-020), ST3Gal II (7275-GT-020), ST3Gal III (10554-GT-020), ST3Gal IV (10496-GT-020), ST3Gal VI (10591-GT-020), ST6Gal I (7620-GT-010), ST6GalNAc I (9154-GT-020), ST6GalNAc II (6468-GT-020), ST6GalNAc IV (6876-GT-010), ST6GalNAc V (6715-GT-020), ST8Sia II (6590-GT-020), ST8Sia IV (7027-GT-020), ST8Sia VI (9687-GT-020).

**Table 1.** Summary of the optimum sialylation conditions for each recombinant sialyltransferase used, and the enzymatic specific activity (ESA) obtained under these conditions, as described in reference [20]

Family	Enzyme	Deletion (aa position)	Quantity (ng)	Time (h)	Concentration CMP-Neu5Al (µM)	ESA^a^ (U/mg)
ST3GAL	ST3GAL1	57-340	4	1	20	55 000 (59 000; 51 000)
	ST3GAL2	52-350	8	1	20	16 470 (16 470; 16 470)
	ST3GAL3	29-375	40	1	10	3 700 (4200; 3200)
	ST3GAL4	41-333	100	4	100	350 (390; 300)
	ST3GAL6	40-331	100	1	100	1250 (1200; 1300)
ST6GAL	ST6GAL1	44-406	4	1	40	91 500 (89 000; 94 000)
ST6GALNAC	ST6GALNAC1	36-600	200	2	50	600 (650; 550)
	ST6GALNAC2	28-373	50	1	40	4260 (4210; 4310)
	ST6GALNAC4	36-302	20	1	50	8500 (9200; 7800)
	ST6GALNAC5	30-336	100	4	100	640 (670; 610)
ST8SIA	ST8SIA2	24-375	100	2	40	1140 (1270; 1010)
	ST8SIA4	40-359	100	2	20	750 (810; 680)
	ST8SIA6	28-398	500	4	20	41 (43; 39)

The optimum conditions of sialylation for each sialyltransferase refer to the best quantity of enzyme, time of sialylation and CMP-Neu5Al concentration to achieve the highest ESA.

a ESA values were determined in duplicate under reported optimal reaction conditions. Data are presented as the Mean accompanied by individual replicate values (Replicate 1; Replicate 2). All values have been systematically rounded to reflect experimental precision in accordance with scientific reporting standards.

Equipment set-up

- 96 well microplate—Thermo Scientific™—F8 Maxisorp loose Nunc-Immuno module (Reference: 469949).
- Spectrophotometer—SpectroStar Nano, BMG Labtech

Buffer protocols/recipes

- NaHCO_3_ buffer (20 mM, pH 9.6; 250 ml): weigh and dissolve 0.42 g NaHCO_3_ in 200 ml water. Adjust pH to 9.6 using 1 M NaOH solution and then make up the volume to 250 ml using water.
- Sodium cacodylate buffer pH 6.4 (100 mM sodium cacodylate, 0.2% Triton CF-54, 10 mM MnCl_2_; 100 ml): weigh 5.35 g sodium cacodylate and dissolve in 80 ml water. Add 500 µl Triton-CF-54 and adjust pH to 6.8 using 1 M HCl. Weigh 0.5 mg MnCl_2_, dissolve in buffer and then adjust pH to 6.4. Make up the volume to 100 ml with water. It is recommended to store the sodium cacodylate buffer at 4°C once prepared and check and readjust the pH before use.
- BSA oxidized: dissolve 1 g of BSA in 20 ml periodic acid and incubated at room temperature for 6 h with slow stirring. It is then dialyzed with water, freeze dried, and analyzed using GC–MS. It appears ivory white to white and fluffy powder [22]. To prepare 100 ml of the 0.05% oxidized BSA needed for the blocking step, 0.05 g of the BSA oxidized powder is dissolved in 100 ml of NaHCO_3_ buffer.
- Asialofetuin: treat 10 mg bovine fetuin with 1 ml of 2 M acetic acid at 80°C for 3 h, and then dialyze with water and freeze dried. The product formed is analyzed using GC–MS to check for the loss of sialic acid on the glycan.

Methods

*Note: Each protocol is presented for one well and quantities can be adapted to the number of wells required per experiment.*

1. Coating

- Dissolve 400 ng of asialofetuin in 100 µl NaHCO_3_ buffer (per well). This mixture is used to coat the microplate wells. Depending upon the number of wells needed per experiment, the quantity and volume may be scaled up.
- Leave the coated microplate at room temperature overnight. Note that a minimum of 8 h is required. In addition, the coated plate could be stored at 4°C up to one week.
- The following morning, remove the asialofetuin and buffer in excess by inverting the microplate carefully to drain the solution.
- Washed the wells with PBST (2 times with 125 µl) followed by PBS (1 time with 125 µl).

2. Blocking

- Add 100 µl of 0.05% oxidised BSA (in NaHCO_3_ buffer) per well and leave at room temperature for 1 h.
- Remove the reagent by gently inverting the plate and wash the wells with PBST (2 times with 125 µl) followed by PBS (1 time with 125 µl).

3. Sialylation

- To prepare a 100 µl reaction mixture for 1 well, mix 5 µl of 800 µM CMP-Neu5Al and 55 µl of 1.8 nM ST6GAL1 in 40 µl 2.5× sodium cacodylate buffer pH 6.4. In the prepared reaction mixture, this corresponds to 40 µM CMP-Neu5Al and 1 nM ST6GAL1 in 1× sodium cacodylate buffer pH 6.4. The reactions are made in triplicate and scaled according to the experiment.
- Incubate the microplate at 37˚ C for 1 h.
- Remove the reaction mixture by gently inverting the microplate and wash the wells with PBST (2 times with 125 µl) followed by PBS (1 time with 125 µl).
  Precaution: While the sialylation reaction duration and enzyme concentration can be varied, deviating from the optimized conditions risks compromising reaction yields (refer to Table 1 for optimum conditions).
  Precaution: While assaying inhibitors against STs, the inhibitor (at desired concentration) is added to the sialylation reaction mixture.

4. Detection

4.1. Bioorthogonal labeling

- For one well (100 µl), mix 300 µM CuSO_4_, 600 µM BTTAA, 2.5 mM sodium ascorbate, and 250 µM azido-biotin and complete with PBS up to a volume of 100 µl. Add the solution to the wells.
- Incubated the plate at 37°C for 1 h.
- After 1 h, remove the reagent by gently inverting the plate and wash the wells with PBST (2 times with 125 µl) followed by PBS (1 time with 125 µl).

4.2. Antibody

- Add 100 µl of anti-biotin antibody conjugated with HRP (1/15000) made in 1× PBST to each well and incubate at 37°C for 1 h.
- After 1 h, remove the reagent by gently inverting the plate and wash the wells with PBST (2 times with 125 µl) followed by PBS (1 time with 125 µl).

5. Measurement

- Add 100 µl of TMB reagent to each well and incubate the reaction in the dark at room temperature.
- At minute 20, read the absorbance at 620 nm using a spectrophotometer.
- Note: All the reactions are colorless except for the TMB reaction. We did not observe any precipitate in the reaction wells, except when we used an old batch of enzyme that had denatured and lost activity.

6. GraphPad analysis

From the absorbance reading, the enzyme activity, specific enzyme activity, apparent K_M_, and apparent V_max_ may be calculated and plotted as a Michaelis–Menten curve using GraphPad Prism version 11.0.2.

## Results

### In-situ ^31^P NMR monitoring of CMP-Sia chemoenzymatic synthesis

At the onset of the reaction (*t* = 0), the ^31^P NMR spectrum of the reaction mixture (1 eq CTP, 1 eq Sia derivative, MgCl_2_ in Tris–HCl buffer, pH 8.5) displays three signals at δ approximately –5.5, –10.5, and –19.5 ppm, corresponding to the three phosphate groups of CTP (Fig. 2). Upon addition of CMP-sialic acid synthetase (CSS) and inorganic pyrophosphatase to initiate the enzymatic reaction, the intensities of these CTP resonances steadily decreased. Concurrently, two new peaks emerged: one at δ approximately 2.5 ppm, attributed to inorganic phosphate (Pi) released into the Tris buffer, and another at δ approximately –4.5 ppm, corresponding to the formation of CMP-SiaNAl. Additionally, a broad, transient signal appeared around δ approximately –5.5 ppm (partially overlapping the original γ-phosphate resonance of CTP), which was assigned to the pyrophosphate (PPi) by-product; this PPi peak vanished over time as it was hydrolyzed by the pyrophosphatase. By the end of the reaction, only the CMP-SiaNAl and Pi signals remained in the spectrum, indicating that CTP had been completely consumed. The reaction mixture was then aliquoted and frozen for future use.

**Figure 2:**
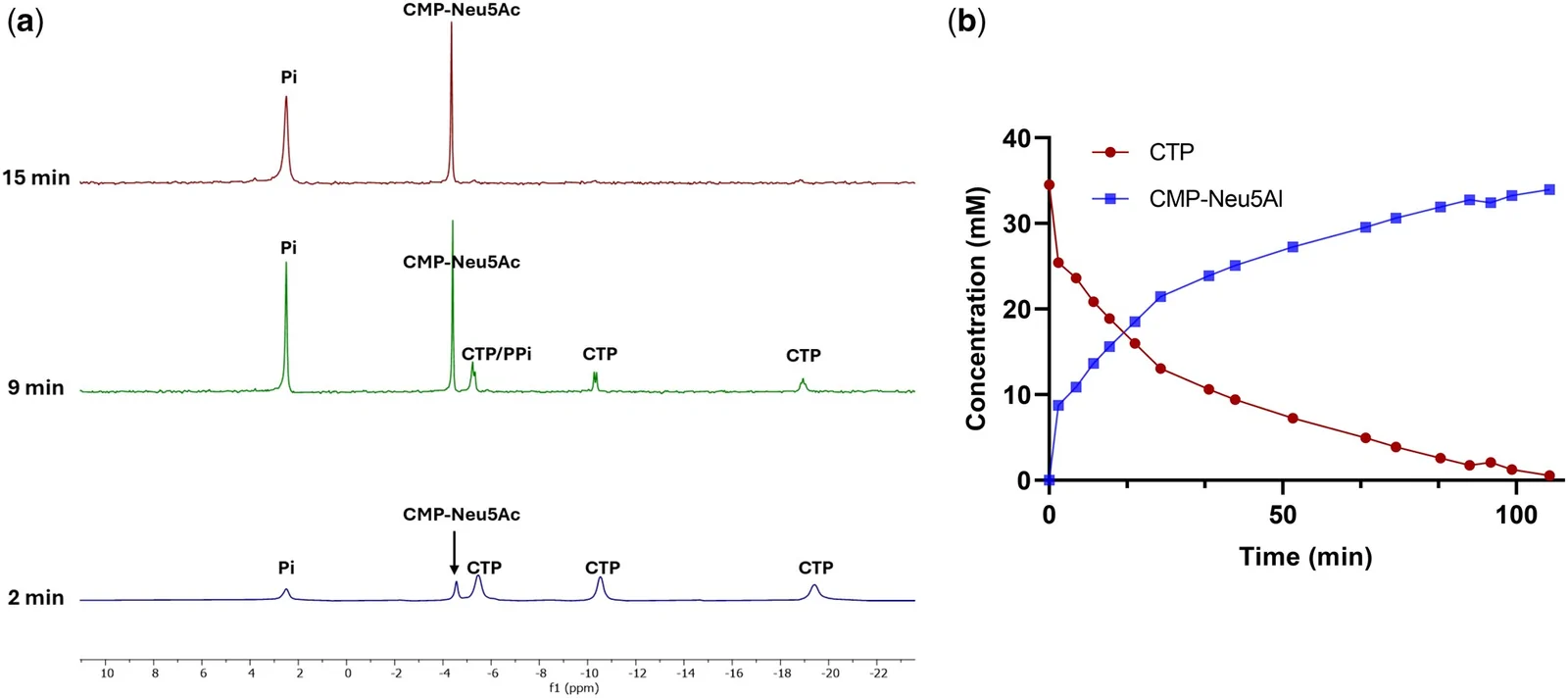
^31^P NMR Monitoring of the chemo-enzymatic synthesis of CMP-Neu5Al in Tris–HCl buffer at 310 K. (a) NMR spectra of key time points of the reaction. (b) Quantitative evolution of CTP (depletion) and CMP-Neu5Al (synthesis). After 110 min, the spectrum shows a reaction yield of 98% based on the peak integration of consumed CTP.

Under these experimental conditions, external referencing using 85% H_3_PO_4_ (as is common in ^31^P NMR) is not possible because of the need for Tris–HCl pH 8.5 buffer. However, the resonance at δ ∼2.5 ppm was independently confirmed in separate control experiments under the same conditions as inorganic phosphate Pi, a value more shielded than usual but consistent with previous reports using similar buffer conditions [23]. The offset is presumably due to specific interactions of Tris^+^ ions with Pi, modifying the electronic environment around the phosphorus nucleus. This signal was subsequently used as the internal chemical shift reference for all spectra.

### MPSA

MPSA (Fig. 3) was carried out to study the activity of the enzyme recombinant human ST6GAL1, using increasing concentrations of the home-made donor substrate, CMP-Neu5Al, and acceptor substrate, asialofetuin (in saturating conditions). The absorbance obtained is converted into activity using the Beer–Lambert equation and Michaelis–Menten plot and Lineweaver–Burke plot were made using GraphPad Prism software. With increasing concentrations of the donor substrate CMP-Neu5Al, the activity of the enzyme increases; however, beyond 40 µM of the donor, the enzyme activity saturates, as is expected in a Michaelis–Menten kinetics model (Fig. 4). The apparent V_max_ (V_max_ app.) of the enzyme was found to be 0.3767 µmol/min/ml and the apparent K_M_ (K_M_ app.) was determined as 7 µM. In comparison with the activities of other mammalian STs, (also determined using the MPSA) the K_M_ app. of ST6GAL1 toward the donor is lower than that of other STs such as ST3GAL4 and ST6GALNAC4, while it is higher than that of STs including ST3GAL1, ST3GAL2, ST3GAL6, ST6GALNAC1, and ST8SIA4 [20]. In terms of the V_max_ app., ST6GAL1 exhibits one of the highest velocities, compared to other STs [20].

**Figure 3:**
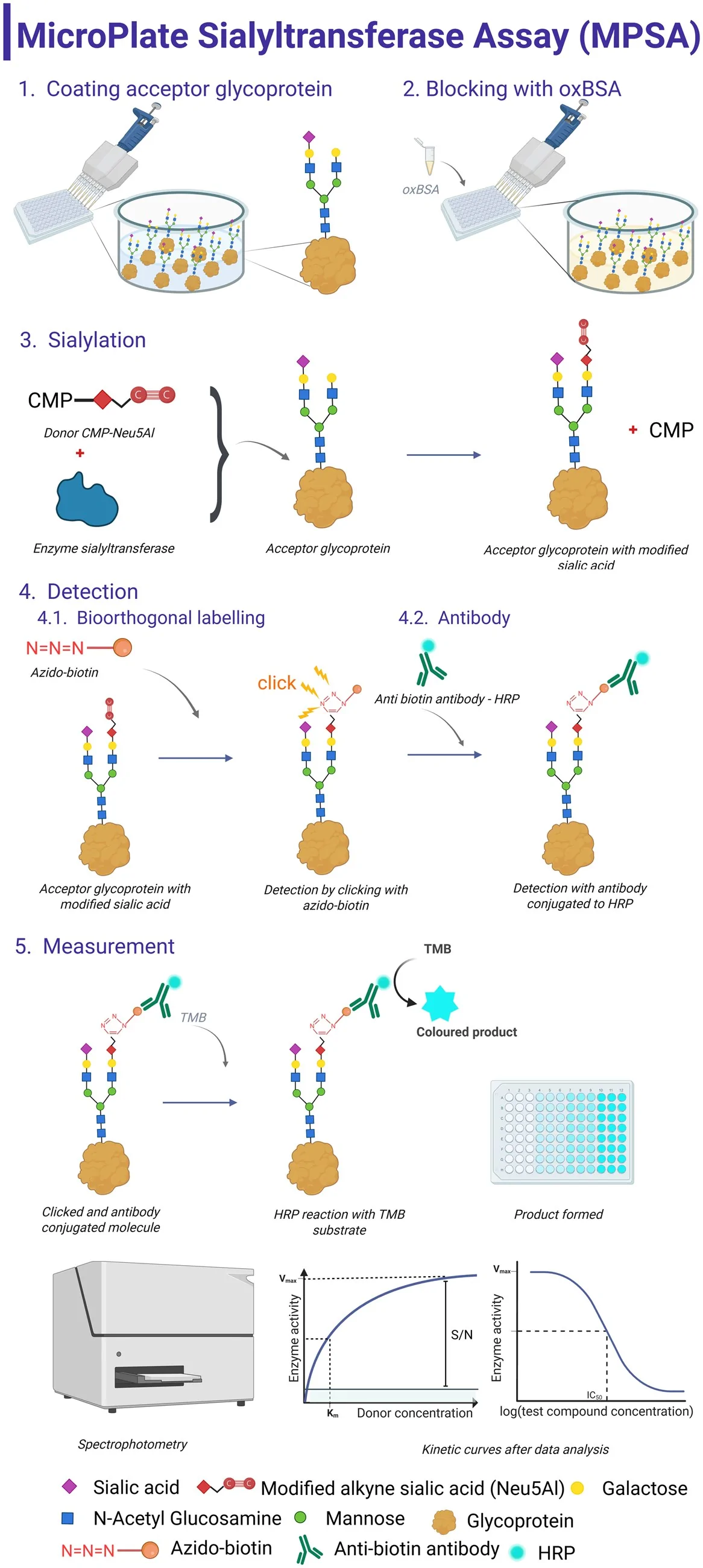
Illustration showing the various steps of the MPSA. 1. The acceptor glycoprotein is coated onto the microplate surface using a multichannel pipette. This step is allowed overnight at room temperature. 2. Blocking the surface using oxidized BSA. 3. Sialylation using the functionalized donor CMP-Neu5Al and the enzyme source leads to transfer of the Neu5Al (red diamond) onto the glycoprotein acceptor. 4. Detection of the transferred Neu5Al is done by first (4.1.) performing biorthogonal labelling with an azido-biotin probe, and second (4.2.) by adding an antibody against biotin. 5. The biotinylated-clicked-Neu5Al product on the acceptor glycoprotein is measured by using reagent TMB that reacts with the antibody-HRP to give a blue product that may be detected at 620 nm. This reading may be converted into enzyme activity using the Beer–Lambert law, and consequently enzyme kinetics may be studied. The schematic graph illustrates the Michaelis–Menten curve obtained, apparent kinetics parameters V_max_ and K_M_, as well as background noise (gray) and signal-to-noise (S/N) ratio. The inhibition by an inhibitor/test compound may also be screened and characterized using MPSA and also dose-response curves may also be constructed to determine the IC50. The illustration was created with BioRender.com (agreement number ZL2A5BOK8E).

**Figure 4:**
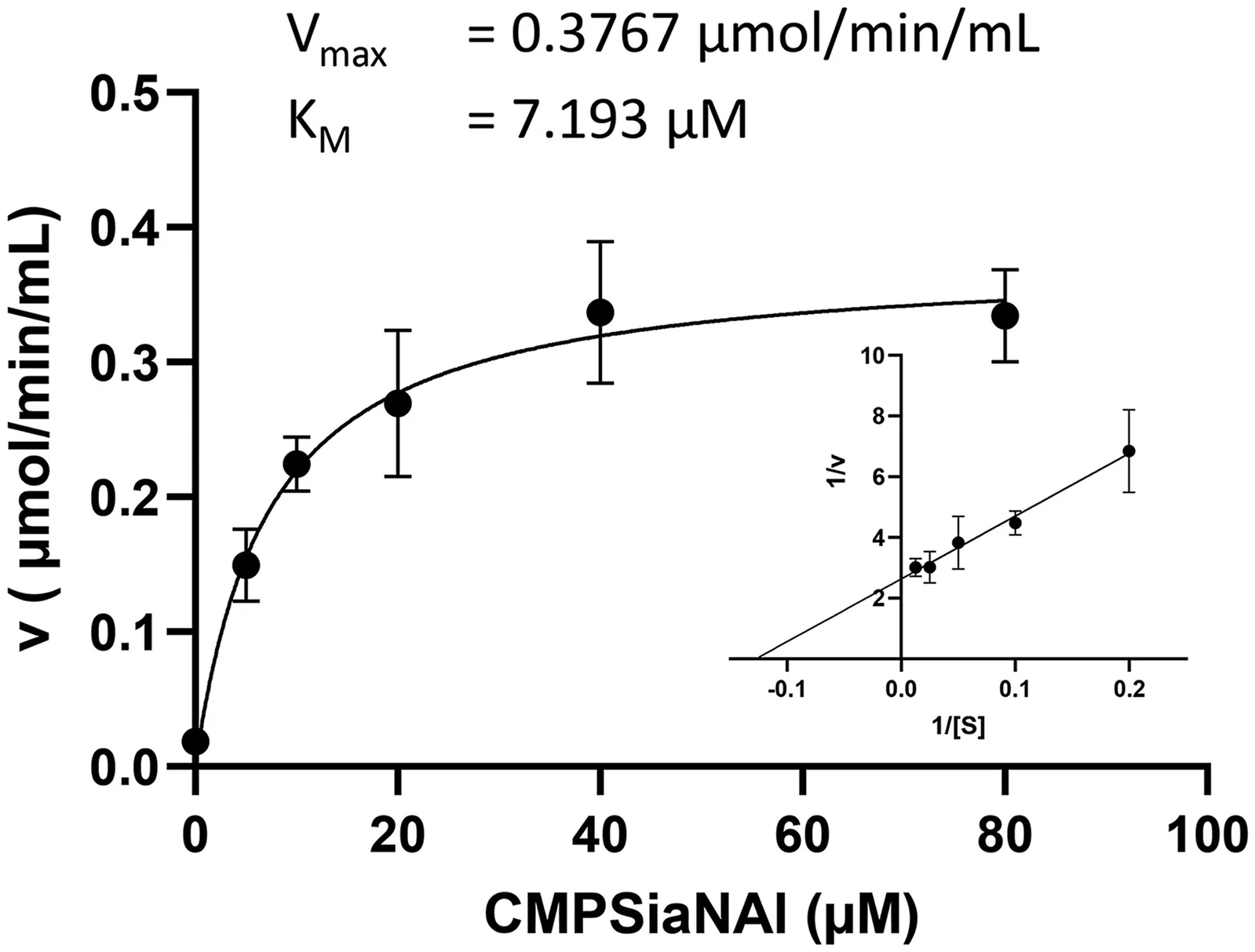
Michaelis–Menten curve for assessment of ST6GAL1 apparent K_M_ for CMP-Neu5Al. The graph shows initial reaction rate of ST6GAL1 across increasing concentrations of donor CMP-Neu5Al and the Lineweaver–Burke plot, from reference [20]. Each concentration was tested in triplicate; error bars represent standard deviation (*n* = 3). Raw data are shown in Supplementary Fig. S4 to get an idea of the dynamic range (0.05 ± 0.01 to 1.2 ± 0.1) and signal-to-noise ratio of the ST6GAL1 assay.

The MPSA was also used to study the inhibition of ST6GAL1 by the well-known sialyltransferases competitive inhibitor CMP [13, 20], illustrated in Fig. 5. Here, 4 ng ST6GAL1 was used and the inhibition by increasing concentrations of CMP was tested with donor concentration at 2× the apparent K_M_ value. With increasing concentration of the inhibitor, we observe a sharp decline in the enzyme activity. The MPSA can thus be used to study the inhibition kinetics, as well. The mode of inhibition and the kinetics parameters and IC_50_ of the inhibitors may be determined also.

**Figure 5:**
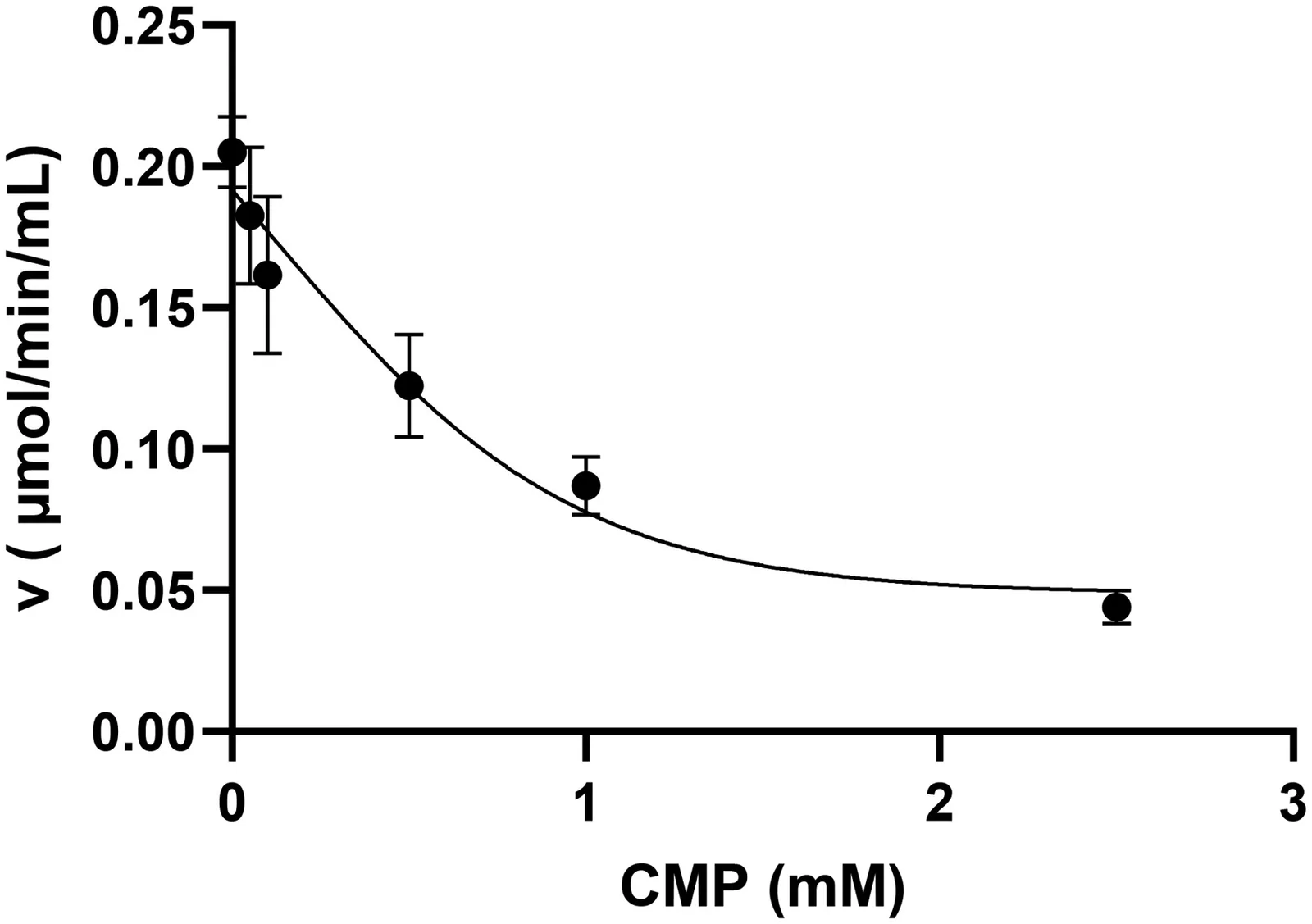
Assessing the effect of inhibitor CMP against the activity of ST6GAL1. The acceptor asialofetuin was coated on the microplate. Sialylation reaction was carried out for 1 h with 4 ng ST6GAL1 and 10 µM of CMP-Neu5Al (i.e. 2× the apparent K_M_ value). The inhibition of ST6GAL1 activity was obtained with increasing concentrations of CMP ranging from 0 to 2.5 mM. Each concentration was tested in triplicate, error bars represent standard deviation (*n* = 3).

## Discussion

Real-time ^31^P NMR monitoring is a routine technique available on most standard NMR spectrometers ensuring rapid, consistent, and highly reproducible synthesis of modified CMP-Sia substrates. This approach is highly straightforward—often simpler to interpret than standard ^1^H-NMR, making it easily accessible to non-specialists. Notably, full kinetic monitoring of CMP-Sia formation is unnecessary; acquiring a few intermittent ^31^P spectra throughout the reaction sequence is sufficient to verify complete conversion.

The modular nature of this synthetic framework allows for straightforward chemical modification and functional diversification of CMP-sialic acid substrates (Supplementary Fig. S3). For instance, Neu5Al or Neu5Az bears a small chemical moiety, enabling downstream detection following metabolic or enzymatic incorporation *via* complementary probes (either lectins, fluorophores, or antibodies) through bioorthogonal click chemistry, which underscores the broad versatility of the strategy. By bypassing substrate instability and labor-intensive chromatographic purification procedures, this integrated protocol establishes a robust and accessible platform for studying ST activity.

The utility of pre-labeled C5/C9 CMP-Sia analogs—such as CMP-BODIPY-Sia [24]—is often limited to enzymes with broad substrate tolerance (e.g. select bacterial STs or mammalian ST6GAL1). Consequently, a major advantage of the MPSA strategy is that functional tags (biotin or fluorophores) are appended post-sialylation, circumventing enzyme incompatibility [25]. Besides, fluorescein-derived CMP-Sia tracers used in alternative fluorescence polarization-based ST assays [26] are notoriously pH-sensitive [27]. The MPSA circumvents these artifacts using standard, accessible microplate technologies for the direct detection of sialylated products, eliminating the need for extensive post-reaction purifications like HPLC or mass spectrometry. In terms of sensitivity, MPSA is fully comparable to the radioactive assays using CMP-[^14^C]Neu5Ac [25], which were historically used to determine fundamental kinetic parameters (i.e. Michaelis–Menten constant K_M_ and V_max_) [15, 28, 29]. However, unlike these early radiometric methods—which require expensive reagents, specialized scintillation instruments, and strict radioactive waste management protocols—the MPSA requires only a standard luminescence microplate reader. In addition, molecular dynamics simulations for human ST8SIA4 demonstrated that the enzyme accommodates this non-naturally modified donor slightly better than the native CMP-Neu5Ac [20] supporting the idea that there are no major differences in sialyltransferase efficiencies, although this remains to be established for all the human STs. Furthermore, the MPSA is highly robust against complex sample matrices. It can accommodate diverse enzyme sources, including both purified recombinant enzymes and unpurified lysates. This is a critical limitation of coupled-enzyme systems (such as the Promega UMP/CMP-Glo assay or malachite green colorimetric phosphatase assays), which measure secondary products like free CMP or inorganic phosphate [16, 30]; such indirect assays are highly prone to background interference from crude biological samples.

Crucially, the MPSA generates quantitative data that permits the accurate calculation of initial reaction rates and enzyme kinetic parameters under uniform reaction conditions (Table 1). The assay represents a universal platform with an exceptional dynamic range and signal-to-noise ratio. For ST6GAL1, background absorbance at 620 nm was 0.05 ± 0.01, reaching a maximum signal of 1.2 ± 0.2. While this response depends on the specific enzyme and batch, the large signal window enables robust kinetic modeling and precise fitting to the Michaelis–Menten equation (raw absorbance data for ST6GAL1 are provided in Supplementary Fig. S4). It remains robust even when evaluating enzymes with low specific activities, such as ST8SIA6, thereby enabling the definition of standardized and reproducible kinetics for any ST acting on *N*- and *O*-glycoproteins [20]. While this protocol highlights asialofetuin as a universal glycoprotein acceptor for determining ST kinetics, several other acceptors—such as ALCAM or PGM—have been successfully paired with MPSA to assess ST substrate specificity [18–20]. Notably, because these glycoproteins feature distinct glycosylation sites, they may yield varying kinetic parameters across different STs.

Because the assay requires minimal enzymatic material (e.g. 4 ng of ST6GAL1), it is exceptionally well-suited for HTS formats. This efficiency opens new avenues for small-molecule inhibitor profiling, facilitating the determination of rigorous inhibitory parameters such as IC_50_ and the thermodynamic inhibition constant Ki for each STs.

The versatility of this protocol allows researchers to investigate STs across diverse biological phyla—ranging from bacteria to mammals—within various physiological contexts or in the presence of targeted inhibitors [31]. Its operational simplicity and minimal hands-on time ensure that this glyco-analytical method is readily exploitable by both specialist glycoscience laboratories and broader biomedical research groups. Future iterations of this protocol may involve automation and further miniaturization to interface with glycan microarrays, as well as integration with complementary analytical readouts like mass spectrometry and advanced fluorescence metrics.

## Limitations

The MPSA setup is ideal for larger glycoproteins (like asialofetuin) that bind well to microplate plastics. However, it is poorly suited for small oligosaccharides, free glycans, or highly hydrophobic glycolipids, which do not coat microplates effectively without specialized plate chemistry. In addition, in MPSA, the acceptor glycoprotein is immobilized on the bottom of a microplate. Immobilization can restrict the flexibility of the protein, meaning certain glycan acceptor sites may become structurally hidden or sterically hindered from interacting with the ST.

## Supplementary Material

bpag058_Supplementary_Data

## Data Availability

This article analyzes existing, publicly available data. No original code was reported in this article. Any additional information required to reanalyze the data reported in this article is available from the lead contact upon request. Reagents generated in this article are available from the lead contact with a completed Materials Transfer Agreement.
