# Supplementary material for: An integrated workflow for real-time chemoenzymatic CMP-sialic acid preparation and a bioorthogonal microplate sialyltransferase assay (MPSA)
Source: Biol Methods Protoc. 2026 Oct 14;11(1):bpag058. doi: 10.1093/biomethods/bpag058 (PMC13722285; doi:10.1093/biomethods/bpag058)
Supplement: Supplementary file 1 [file bpag058_supplementary_data.docx]

**Supporting Information**

**An Integrated Workflow for Real-Time Chemoenzymatic CMP-Sialic Acid Preparation and a Bioorthogonal Microplate Sialyltransferase Assay (MPSA)**

Lucas Lancel^1,+^, Sushmaa Dangudubiyyam^1,+^, Clémence Simon^1^, Hugo Segura^1^, Christophe Biot^1^, Yann Guérardel^1^, Anne Harduin-Lepers^1,*^, Cédric Lion^1,*^

^1^ Univ. Lille, CNRS, UMR 8576 - UGSF - Unité de Glycobiologie Structurale et Fonctionnelle, Lille, France

^+^ equal contribution, co-first authors

^*^ correspondence: UGSF, Bât. C9, avenue Mendeleiev, Cité Scientifique, 59650 Villeneuve d’Ascq. Email: [anne.harduin-lepers@univ-lille.fr](mailto:anne.harduin-lepers@univ-lille.fr) ; [cedric.lion@univ-lille.fr](mailto:cedric.lion@univ-lille.fr)

Supplemental figures S1-S5, pages 2-5

Supplemental data NMR spectra, pages 6-8

**Supplementary figure S1**: A) Thin layer chromatography analysis of succinimidyl 4-pentynoate synthesis, eluant cyclohexane/ethyl acetate (7:3) either revealed with bromocresol green (left) to notice carboxylic group or with UV (right). B) TLC of N-pentynoyl mannosamine synthesis, eluant propan-1-ol / 20% ammonia / H_2_O (6 : 1 : 2.5) revealed with vanillin. C) TLC of Neu5Al synthesis, eluant propan-1-ol / 20% ammonia / H_2_O (6 : 1 : 2.5) and revealed with orcinol. CS = cospot, RM = reaction medium.

**Supplementary figure S2.** Chromatograms of Neu5Al purification, detected with (A) Evaporative Light Scattering Detection or (B) UV at 215 nm.

**Supplementary Figure S3**. ^31^P NMR monitoring of the chemo-enzymatic synthesis of natural (CMP-Neu5Ac, CMP-Neu5Gc, CMP-Kdn) and chemically modified (CMP-Neu5Az) CMP-sialic acid derivatives. Neu5Ac: N-acetylneuraminic acid; Neu5Gc: N-glycolylneuraminic acid; Kdn: 3-deoxy-non-2-ulosonic acid; Neu5Az: N-azidoacetylneuraminic neuraminic acid.

**Supplemental figure S4**: Raw data of ST6GAL1 enzymatic activity measured with MPSA at various concentrations of CMP-sialic acid donor corresponding to figure 4 showing absorbance at 260 nm obtained for various concentration of the CMP-sialic acid donor. Background noise is about 0.05 +/- 0.01 and signal to noise ratio is 1.3 / 0.05 for ST6GAL1.

**NMR spectra of succinimidyl 4-pentynoate**

**1H NMR (300 MHz, CDCl_3_) :** δ 2.92 (t, J = 7.3 Hz, 2H), 2.84 (s, 4H), 2.62 (tdd, J = 7.3, 2.7, 0.6 Hz, 2H), 2.05 (t, J = 2.5 Hz, 1H).

**13C NMR (75 MHz, CDCl_3_):** 168.86 (CH), 167.00 (CH), 70.03 (CH), 30.32 (CH_2_), 25.57 (CH_2_, C), 14.10 (CH)

^1^H NMR spectrum

^13^C NMR spectrum

**NMR spectra of *N*-4-pentynoyl-D-mannosamine (ManNAl)**

**α ^1^H NMR (300 MHz, D_2_O)**: δ 5.00 (d, *J =* 1.4 Hz, 1H, Hα), 4.90 (d, *J =* 1.6 Hz, 1H, Hβ), 4.35 (dd, *J =* 4.4, 1.4 Hz, 1H, Hβ), 4.23 (dd, *J =* 4.7, 1.4 Hz, 1H, Hα), 3.93 (dd, *J =* 9.8, 4.7 Hz, 1H, Hα), 3.80 – 3.62 (m, 6H, Hα + Hβ), 3.50 (t, *J =* 9.6 Hz, 1H, Hα), 3.40 (t, *J =* 9.8 Hz, 1H, Hβ), 3.29 (ddd, *J =* 9.9, 4.8, 2.3 Hz, 1H, Hβ), 2.58 – 2.32 (m, 8H, Hα+ Hβ), 2.26 (t, *J =* 2.3 Hz, 2H, Hα + Hβ).

**^13^C NMR (75 MHz, D_2_O)**: δ 175.98 (Cβ), 175.26 (Cα), 93.17 (Cα), 92.84 (Cβ), 83.85 (Cβ), 83.43 (Cα), 76.28 (Cβ), 71.96 (Cβ), 71.94 (Cα), 70.14 (Cα), 69.99 (Cβ), 68.73 (Cα), 66.72 (Cα), 66.45 (Cβ), 60.36 (Cα + Cβ), 53.98 (Cβ), 53.15 (Cα), 34.25 (Cβ), 34.10 (Cα), 14.40 (Cα), 14.26 (Cβ).

^1^H NMR spectrum

^13^C NMR spectrum

**NMR spectra of synthetically modified *N*-pentynoyl neuraminic acid (Neu5Al)**

**6: SiaNAl (*N*-4-pentynoylneuraminic acid)**

**^1^H NMR (600 MHz, D_2_O)**: δ 3.96 – 3.86 (m, 2H), 3.83 (t, *J =* 10.1 Hz, 1H), 3.72 (dd, *J =* 11.9 Hz, 2.7, 1H), 3.64 (ddd, *J =* 9.2, 6.7, 2.7 Hz, 1H), 3.51 (d, *J =* 9.2 Hz, 1H), 3.49 – 3.43 (m, 1H), 2.47 – 2.34 (m, 4H), 2.28 (d, *J =* 2.0 Hz, 1H), 2.10 (dd, *J =* 13.0 Hz, 4.9, 1H), 1.70 (dd, *J =* 12.7, 11.7 Hz, 1H).

**^13^C NMR (75 MHz, D_2_O)**: δ 175.43, 172.91, 95.16, 83.54, 70.40, 70.37, 70.23, 68.29, 66.45, 63.21, 52.04, 38.79, 34.67, 14.53.

^1^H NMR spectrum

^13^C NMR spectrum
